# Smart Blood Vessel Detection System for Laparoscopic Surgery

**DOI:** 10.1109/JTEHM.2022.3159095

**Published:** 2022-03-11

**Authors:** Ching-Chia Li, Bor-Shing Lin, Sheng-Chen Wen, Yuan-Teng Liang, Hung-Yu Sung, Jhen-Hao Jhan, Bor-Shyh Lin

**Affiliations:** Department of UrologyKaohsiung Medical University Chung-Ho Memorial Hospital 89234 Kaohsiung 80756 Taiwan; Department of Computer Science and Information EngineeringNational Taipei University 63284 New Taipei City 237303 Taiwan; Institute of Imaging and Biomedical PhotonicsNational Yang Ming Chiao Tung University Tainan 71150 Taiwan

**Keywords:** Laparoscopic surgery, near infrared spectroscopy, hemoglobin parameters, neural network, vascular location

## Abstract

Objective: Compared with traditional surgery, laparoscopic surgery offers the advantages of smaller scars and rapid recovery and has gradually become popular. However, laparoscopic surgery has the limitation of low visibility and a lack of touch sense. As such, a physician may unexpectedly damage blood vessels, causing massive bleeding. In clinical settings, Doppler ultrasound is commonly used to detect vascular locations, but this approach is affected by the measuring angle and bone shadow and has poor ability to distinguish arteries from veins. To tackle these problems, a smart blood vessel detection system for laparoscopic surgery is proposed. Methods: Based on the principle of near-infrared spectroscopy, the proposed instrument can access hemoglobin (HbT) parameters at several depths simultaneously and recognize human tissue type by using a neural network. Results: Using the differences in HbT and StO_2_ between different tissues, vascular and avascular locations can be recognized. Moreover, a mechanically rotatable stick enables the physician to easily operate in body cavities. Phantom and animal experiments were performed to validate the system’s performance. Conclusion: The proposed system has high ability to distinguish vascular from avascular locations at various depths.

***Clinical and Translational Impact Statement***—A smart blood vessel detection system for laparoscopic surgery was proposed. Using the differences in HbT and StO_2_ values between different tissues, vascular and avascular locations can be successfully recognized. (Category: Preclinical Research).

## I. Introduction

With the advent of laparoscopy, patients now experience less pain at a surgical site after the surgery. Compared with open surgery, laparoscopy offers a better cosmetic effect, quicker recovery, and comparable efficacy. However, laparoscopic procedures require a steeper learning curve than open procedures. In open surgery, the surgeon must confirm a vessel’s location through visual inspection and palpation. However, blood flow pulsation cannot be observed through palpation—the surgeon depends on their experience and visual inspection; thus, injuries such as massive bleeding due to accidental vessel rupture may occur during laparoscopic surgery.

In laparoscopic surgery, Doppler ultrasound is generally used to determine the location of blood vessels. The Doppler instrument [1] uses the frequency difference between the transmitted and reflected ultrasound signals to estimate blood flow change and determine the possible location of blood vessels. However, the tip direction of the ultrasound probe is difficult to control when the probe is passed through a trocar in the human body [2], and the quality of ultrasound imaging is lower at greater depth [3]. Moreover, Doppler ultrasound can only distinguish a change in blood flow but not distinguish between tissue types [4]. In 2007, Klein *et al.* designed a piezoelectric needle producing 1–8-kHz vibrations at the needle tip to assist the Doppler instrument in monitoring blood flow [5]. To improve the quality of color Doppler images, the needle must be inserted into the human body to vibrate the local tissue. Recently, the near-infrared vein visualization device was developed for real-time visualization of superficial vessels under the skin [6]–[8]; near-infrared light (wavelength: ~785 nm) is used to penetrate the skin and blood vessels and is partially absorbed at avascular locations but not at vascular locations. Using the difference in absorption between the vascular and avascular locations, the device determines the location of blood vessels. However, this technique cannot be employed for measurement depths exceeding 5 mm.

To address the aforementioned problems, a smart blood vessel detection system for laparoscopic surgery was proposed in this study for differentiating vascular and avascular locations *in vivo*. Based on the difference in absorption spectra for different tissues [9], [10], the proposed instrument can differentiate vascular and avascular locations at various depths of human tissue simultaneously. Moreover, the rotatable stick enables the physician to easily control the angle and direction of the optical probe in the human body cavity. Phantom and animal experiments were performed to validate the system’s performance; the experimental results confirmed that the proposed instrument has high performance in detecting blood vessels at different depths.

## II. Methods

### A. Design and Implementation of the Smart Blood Vessel Detection System

**FIGURE 1.**
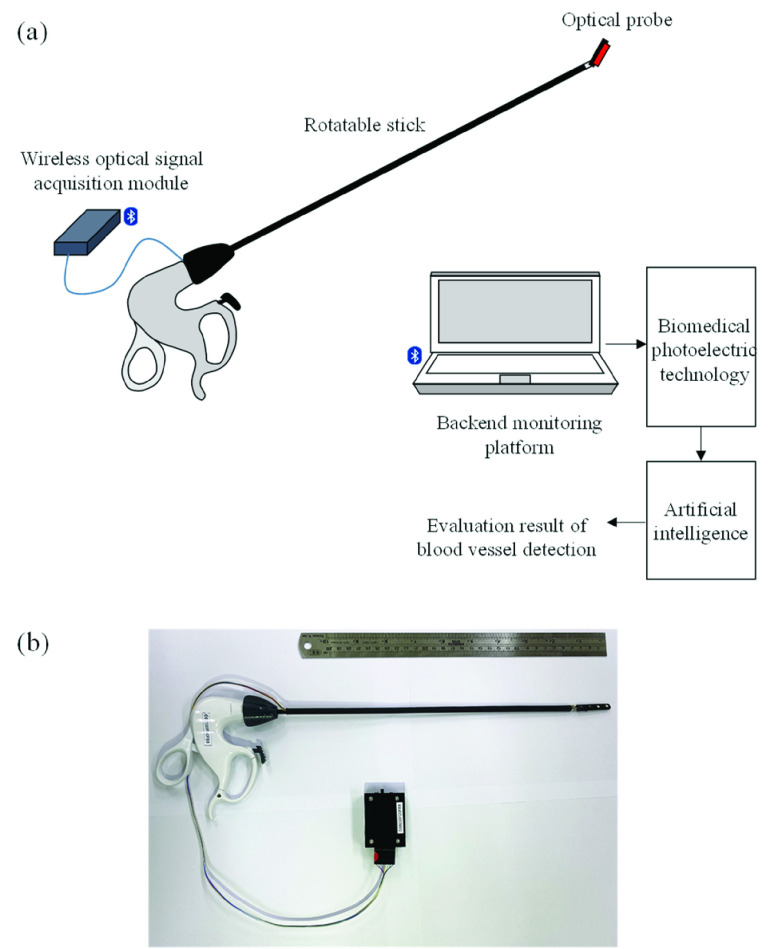
(a) Basic structure and (b) photograph of the smart blood vessel detection system for laparoscopic surgery.

Using the difference in absorption spectra of different human tissues, tissue type can be recognized through the changes in optical intensity at a specific wavelength [11]–[14], and this approach can be employed for detecting blood vessels under adipose or lymphatic tissue. The basic structure and a photograph of the proposed smart blood vessel detection system for laparoscopic surgery are shown in Fig. 1 (a) and Fig. 1 (b), respectively; the proposed system mainly contains an optical vessel detection instrument and a backend monitoring platform.

The optical vessel detection instrument consists of a rotatable stick, an optical probe, and a wireless optical signal acquisition module. The optical probe contains two photodiodes (PDs; PD15–22C; EVERLIGHT, Taipei, Taiwan) and two dual-wavelength light-emitting diodes (LEDs; SMT700/910; Marubeni Corp., Tokyo, Japan). The PDs are used to detect the light signal reflected by human tissue (adipose or lymphatic tissue), whereas the LEDs act as dual-wavelength light sources. When the LED light passes through human tissue, optical attenuation is caused by scattering and absorption. For most components of human tissue, absorption in the near-infrared wavelength region (650–1000 nm) is very small, and oxyhemoglobin (HbO_2_) and deoxyhemoglobin (Hb) are two of the primary absorbers. Therefore, for near-infrared light, the optical attenuation change $\Delta OD\left ({\lambda }\right)$ can be simply expressed as

\begin{align*}&\hspace {-2.4pc}\Delta OD\left ({\lambda }\right)=-log \frac {I_{o}\left ({\lambda }\right)}{I_{i}\left ({\lambda }\right)}=\left ({\varepsilon _{HbO_{2}}^{\lambda }\cdot \Delta \left [{ HbO_{2} }\right]}\right. \\&\qquad \qquad \qquad \qquad \left.{+\,\varepsilon _{Hb}^{\lambda }\cdot \Delta \left [{ Hb }\right] }\right)\cdot L\cdot B\left ({\lambda }\right),\tag{1}\end{align*}

where $I_{o}\left ({\lambda }\right)$ and $I_{i}\left ({\lambda }\right)$ are the light intensity of the incident light and light reflected by the human tissue, respectively; $L$ is the distance between the light source and detector; and $B\left ({\lambda }\right)$ is the modified optical path factor [15] corresponding to the wavelength $\lambda $. The parameters $\varepsilon _{HbO_{2}}^{\lambda }$ and $\varepsilon _{Hb}^{\lambda }$ are the molar extinction coefficients of ${\mathrm {HbO}}_{2}$ and Hb, respectively. The hemoglobin changes $\Delta \left [{ Hb }\right]$ and $\Delta \left [{ HbO_{2} }\right]$ can be estimated from the multiwavelength optical attenuation changes by using the least square approximation method:

\begin{align*} \Delta \left [{ \left ({\begin{array}{cccccccccccccccccccc} \mathrm {Hb}\mathrm {O}_{2}\\ \mathrm {Hb}\end{array} }\right) }\right]=\frac {1}{\mathrm {L}}{(\mathrm {E}^{\mathrm {T}}\cdot \text {E})}^{-1}\cdot \mathrm {E}^{\mathrm {T}}\cdot \text {A},\tag{2}\end{align*}

where

\begin{align*} \mathrm {E}=\left [{ \begin{matrix} \displaystyle \varepsilon _{{\mathrm {HbO}}_{2}}^{\lambda _{1}} &\quad \varepsilon _{\mathrm {Hb}}^{\lambda _{1}}\\[0.5pc] \displaystyle \varepsilon _{{\mathrm {HbO}}_{2}}^{\lambda _{2}} &\quad \varepsilon _{\mathrm {Hb}}^{\lambda _{2}}\\ \displaystyle \end{matrix} }\right],\quad \mathrm {A}=\left [{ \begin{matrix} \displaystyle \frac {\Delta OD\left ({\lambda _{1} }\right)}{\mathrm {B}(\lambda _{1})}\\[0.8pc] \displaystyle \frac {\Delta OD\left ({\lambda _{2} }\right)}{\mathrm {B}(\lambda _{2})}\\ \displaystyle \end{matrix} }\right].\end{align*}

Herein, 640- and 910-nm-wavelength light is used. Thus, the isosbestic point of the absorption spectra of HbO_2_ and Hb is approximately 800 nm. Finally, the change in the total hemoglobin (HbT) concentration ( $\Delta [HbT]$) and tissue oxygen saturation (StO_2_) can be obtained from $\Delta \left [{ Hb }\right]$ and $\Delta \left [{ HbO_{2} }\right]$:

\begin{align*} \Delta [HbT]=&\Delta \left [{ Hb }\right]+\Delta \left [{ HbO_{2} }\right], \tag{3}\\ \mathrm {St}\mathrm {O}_{2}=&\frac {\Delta \left [{ HbO_{2} }\right]}{\Delta [HbT]}\times 100\%.\tag{4}\end{align*}

**FIGURE 2.**
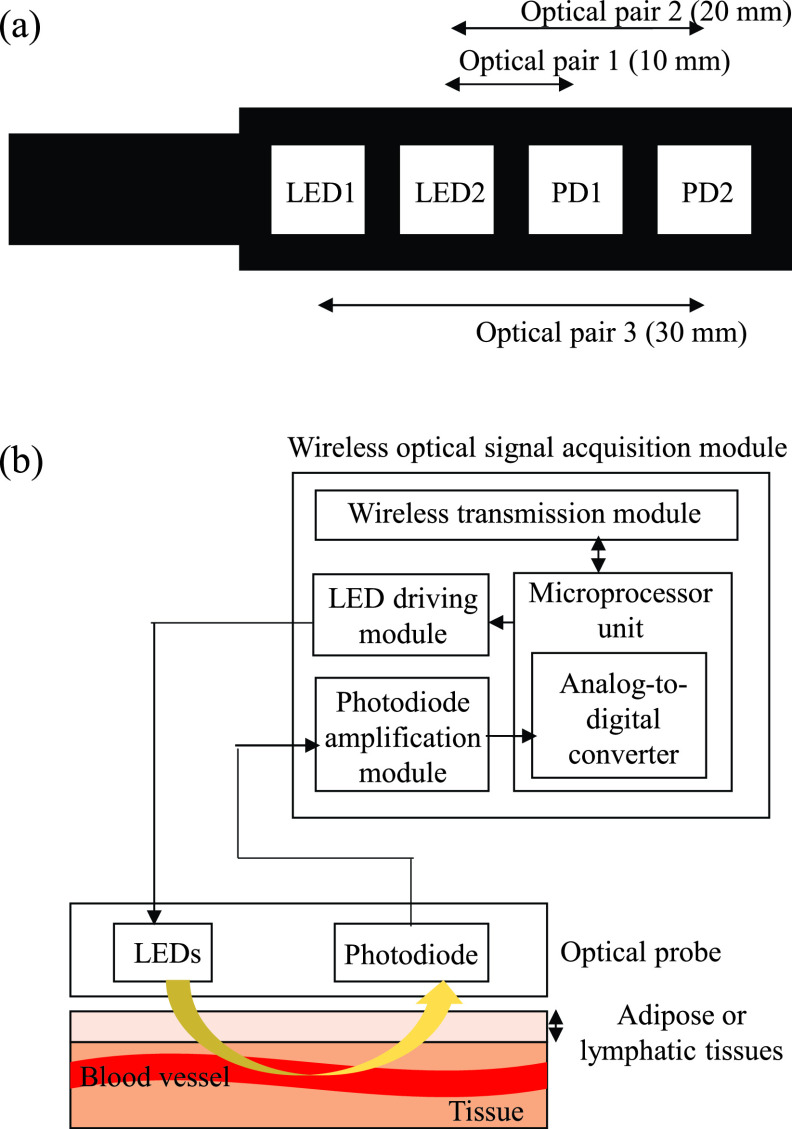
(a) Placement of optical probe, and (b) block diagram of the designed wireless optical signal acquisition module.

Because the penetration depth is approximately half the distance between the light source and detector [16], the optical probe, as shown in Fig. 2 (a), is designed to contain three optical pairs for monitoring blood vessels at different depths simultaneously. The distances between the LEDs and PDs are set as 10 mm (optical pair 1), 20 mm (optical pair 2), and 30 mm (optical pair 3). The optical probe is fixed on the rotatable stick to assist insertion into the body cavity through a trocar. The front end of the rotatable stick can be spun at various angles, thereby enabling the optical probe to easily navigate the curved surfaces of human tissue.

The wireless optical signal acquisition module, illustrated in Fig. 2 (b), contains a microprocessor unit, a wireless transmission module, a photodiode amplification module, and an LED driving module. The LED driving module is designed to drive the LEDs by using the control command of a microprocessor unit (RX210, Renesas Electronics Corp., Tokyo, Japan) to turn on and off the dual-wavelength light source. The light reflected by human tissue is transformed into a voltage signal by the PDs and amplified by the PD amplification module. Next, the received light signal is digitized using the analog-to-digital converter in the microprocessor unit with a sampling rate of 60 Hz and then sent to the wireless transmission module for transmission to the backend monitoring platform.

The backend monitoring platform is designed for the Windows 10 operating system. A real-time monitoring program was developed using Microsoft Visual C# for estimating, displaying, and storing hemoglobin parameters as well as detecting blood vessels.

### B. Classification of Vascular and Avascular Locations

**FIGURE 3.**
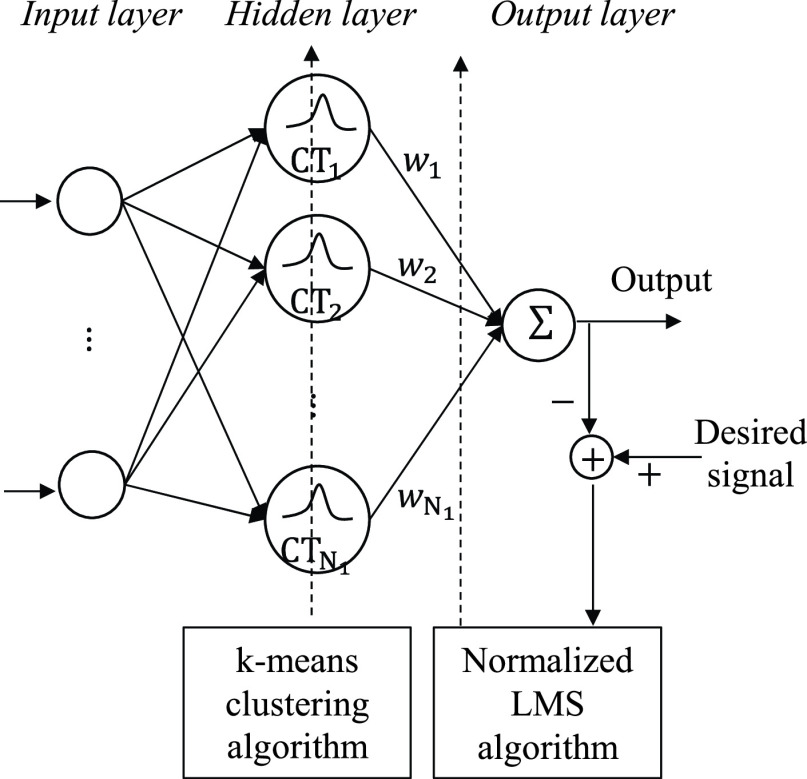
Basic structure of the RBFNN.

The radial-basis-function neural network (RBFNN) [17], [18] is used to classify vascular and avascular locations. The RBFNN is a nonlinear data modeling approach that the hidden neurons can provide the non-linear transform function for the difference between the input vector and the hidden center vector, and the output neuron is contributed by a linear weighted combination of these hidden neuron outputs. The center vectors in the hidden neurons can be viewed as the prototype characteristic vectors taken from the training sets Therefore, it offers the advantages of simple network structure, fast training procedure, and excellent approximation capability. The network structure contains three layers, as illustrated in Fig. 3: an input layer ( $N_{0}$ neurons), a hidden layer ( $N_{1}$ neurons), and an output layer (1 neuron). HbT and StO_2_ are used as the input of the RBFNN. The output $Y(n)$ of the RBFNN at the $n$-th trial input can be expressed as

\begin{equation*} Y\left ({n }\right)=\boldsymbol {Z}^{T}\left ({n }\right)\cdot \boldsymbol {W},\tag{5}\end{equation*}

where $\boldsymbol {W}=\left [{ w_{1},w_{2},\cdots,w_{k},\ldots,w_{N_{1}} }\right]^{T}$ is the weight vector and $w_{k}$ denotes the weight between the $k$-th hidden neuron and output neuron.

$\boldsymbol {Z}\left ({n }\right)=\left [{ z_{1}\left ({n }\right),z_{2}\left ({n }\right),\cdots,z_{k}\left ({n }\right),\ldots,z_{N_{1}}\left ({n }\right) }\right]^{T}$ is the output vector of the hidden neurons. The $k$-th neuron output $z_{k}\left ({n }\right)$ can be calculated using the Gaussian basis function:

\begin{equation*} z_{k}\left ({n }\right)=exp\left ({-\frac {\left \|{ \boldsymbol {I}\left ({n }\right)-{\boldsymbol {CT}}_{k} }\right \|^{2}}{2\sigma ^{2}\left ({n }\right)} }\right),\tag{6}\end{equation*}

where $\boldsymbol {I}\left ({n }\right)$ and $\sigma \left ({n }\right)$ denote the $n$-th trial input vector and its standard deviation respectively, and ${\boldsymbol {CT}}_{k}$ is the center vector of the $k$-th hidden neuron. The operator $\left \|{ \boldsymbol {.} }\right \|$ denotes the Euclid norm between two vectors. A self-organized learning procedure (k-means clustering [19], [20]) and the normalized least-mean-square algorithm [21] are used to train the center vector ${\boldsymbol {CT}}_{k}$ and weight vector $\boldsymbol {W}$, respectively. In the learning stage, the output for the vascular and avascular locations is 1 and 0, respectively. Therefore, if the output of the RBFNN is larger than a given threshold, the corresponding location is classified as vascular; otherwise, it is classified as avascular.

### C. Design of the Animal Experiment

**FIGURE 4.**
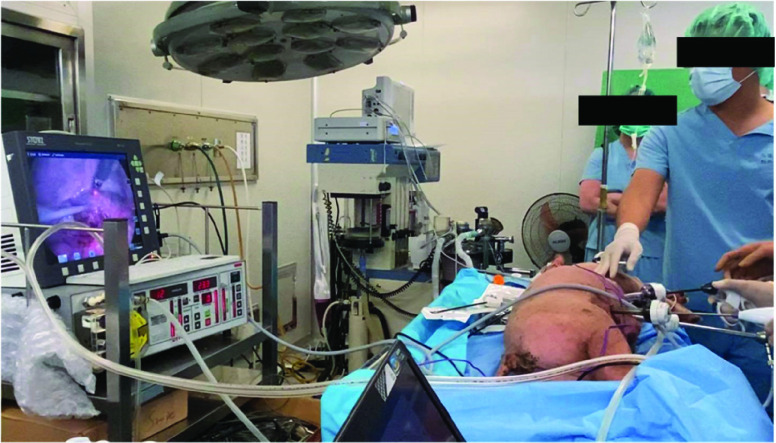
Setup of the animal experiment.

In this study, six duroc Chinese native piglets with an average weight of approximately 21 kg were used. The animal use protocol was reviewed and approved by the Institutional Animal Care and Use Committee (IACUC), Kaohsiung Medical University, Kaohsiung, Taiwan. The IACUC protocol number is 108038. Zoletil IM (10–25 mg/kg) was administered as an intramuscular injection for general anesthesia. The animals were ventilated, intubated, and then maintained with 1%–3% sevoflurane (inhalation anesthetic). The animal experiment was performed as part of the *in vivo* study. Fig. 4 shows the animal experiment wherein the abdomen of the piglet was penetrated using trocars (Lagis, Shielded Bladed Trocar, Taiwan, and ENDOPATH XCEL® Bladeless Trocars, NJ, USA). The proposed smart blood vessel detection system was inserted through the trocar and detected various parts of the body, such as inferior tissue veins, inferior tissue arteries, the liver, and muscles. The proposed system was employed to measure the optical and hemoglobin parameters in different tissue parts, and locations possibly containing blood vessels were determined. Analysis of variance was used to analyze the experimental data, with significance set as p < 0.05.

## III. Experimental Results

### A. Performance in Detecting Blood Vessels in a Phantom

**FIGURE 5.**
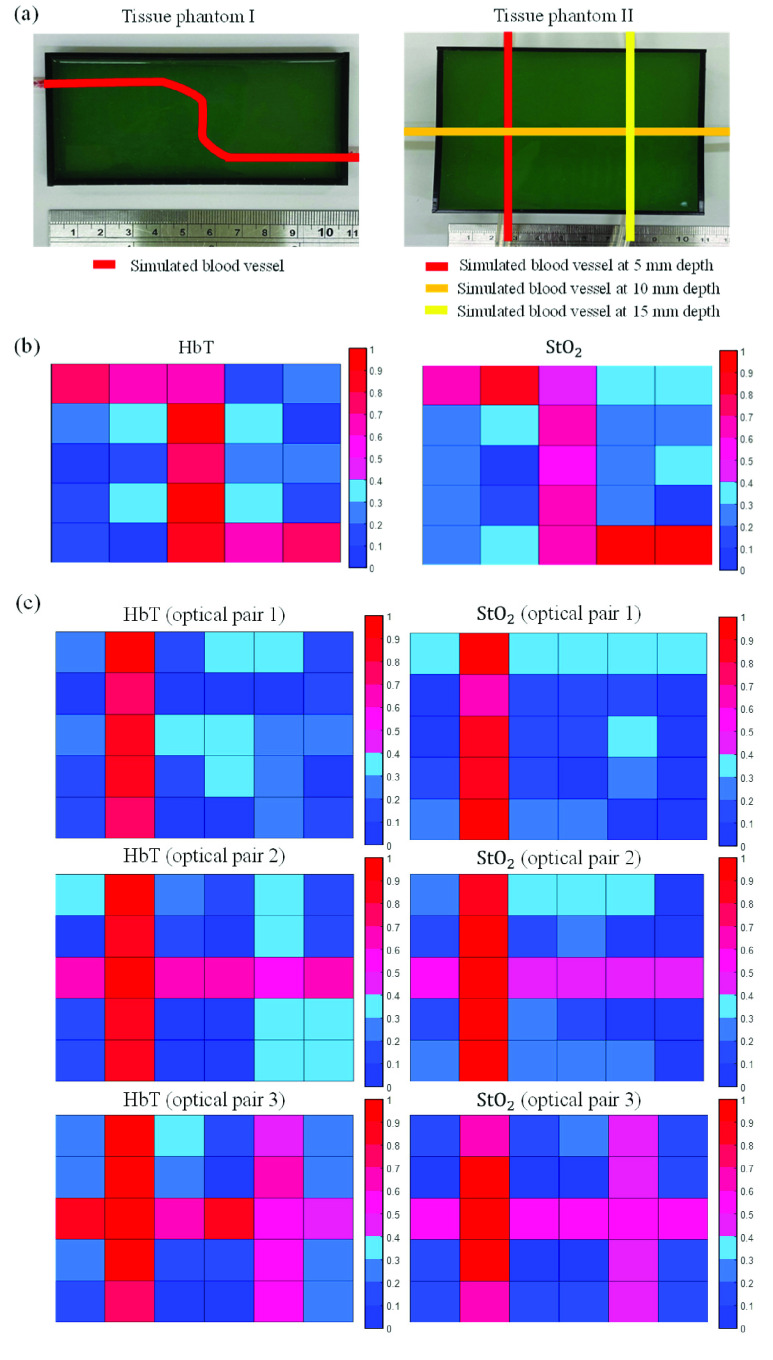
(a) Two types of tissue phantom placement, and measurement results for (b) tissue phantom I and (c) tissue phantom II.

The performance of the proposed system was first validated using a phantom experiment. Two tissue phantoms made of polydimethylsiloxane (PDMS; SC-812, Hisn Han International Trading, New Taipei City, Taiwan) and dye (GY-IR-500, Go Yen Chemical Group, Kaohsiung, Taiwan) were employed. In tissue phantom I (size: 105 mm $\times $ 40 mm $\times $ 30 mm), a plastic tube (inside diameter: 2 mm) was placed at a depth of 5 mm to simulate a blood vessel. In tissue phantom II (size: 115 mm $\times $ 70 mm $\times $ 30 mm), three plastic tubes were placed at depths of 5, 10, and 15 mm to simulate three blood vessels, as illustrated in Fig. 5(a). All of these plastic tubes were filled with red ink to simulate blood. The use of this device has to be operated by hand scanning. Therefore, we divided the area of these phantoms into several parts for scanning test. The minimal scanning partition is about 21 mm $\times $ 8 mm. The experimental results, shown in Fig. 5 (b), indicated that the values of HbT and StO_2_ at the location above the plastic tube in tissue phantom I were higher than at other locations. The values of HbT and StO_2_ estimated by optical pair 1 at depths >5 mm in tissue phantom II were relatively high in Fig. 5(c). However, all HbT and StO_2_ values estimated by optical pair 3 at depths of 5, 10, and 15 mm were also relatively high.

### B. Optical and Hemoglobin Parameters of Different Tissues in the Vivo Experiment

**FIGURE 6.**
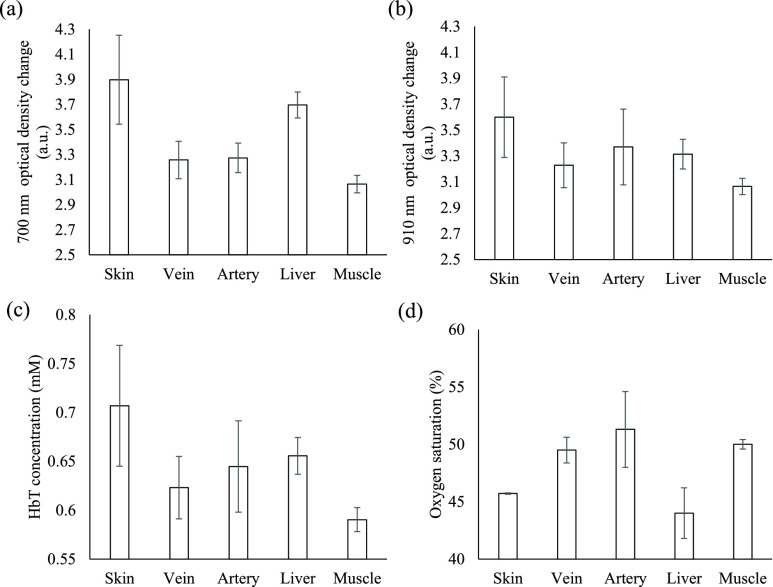
(a) Plot of 700- and (b) 910-nm-wavelength optical density changes, and (c) HbT and (d) StO_2_ values for various tissues.

The optical densities and hemoglobin parameters for different tissues were investigated in the animal experiment. Fig. 6 (a) and Fig. 6 (b) show the optical density changes in various tissues corresponding to the wavelengths 700 and 910 nm, respectively. At 700 nm, the change in optical density for veins was slightly smaller than that for arteries, but at 910 nm, the change in the optical density for veins was much smaller. For the 700-nm wavelength, the optical density change was greatest for the liver. The change in the optical density was smallest for muscle at both 700 and 910 nm. The differences in the changes in optical density at 700 nm (p = 0.000) and at 910 nm (p = 0.000) for different tissues were significant.

Fig. 6 (c) and Fig. 6 (d) show the changes in HbT and StO_2_ for various tissues. The HbT values for arteries, veins, and the liver were higher than those for skin and muscle. However, for arteries, veins, and the liver, the StO_2_ value was higher for arteries than veins, and the StO_2_ value for the liver was lowest. The StO_2_ value for muscle was higher than that for skin or the liver, but the muscle HbT value was smaller than that for skin. The differences in HbT (p = 0.000) and StO_2_ (p = 0.000) between the various tissues were also significant.

### C. Performance in Classification of Vascular Versus Avascular Locations

The performance of the RBFNN in classifying vascular and avascular locations was investigated using several binary test parameters. True-positive (TP) means that a vascular group was correctly identified; false-positive (FP), an avascular group was incorrectly identified as a vascular group; true-negative (TN), an avascular group was correctly identified; and false-negative (FN), a vascular group was incorrectly identified as an avascular group. The F-measure—the harmonic mean of recall (sensitivity) and precision (positive predictive value, PPV)—was used to determine the optimal threshold.

\begin{equation*} \mathrm {F\_{}measure} =2\cdot \frac {Recall\cdot Precision}{Recall+Precision}\tag{7}\end{equation*}

**FIGURE 7.**
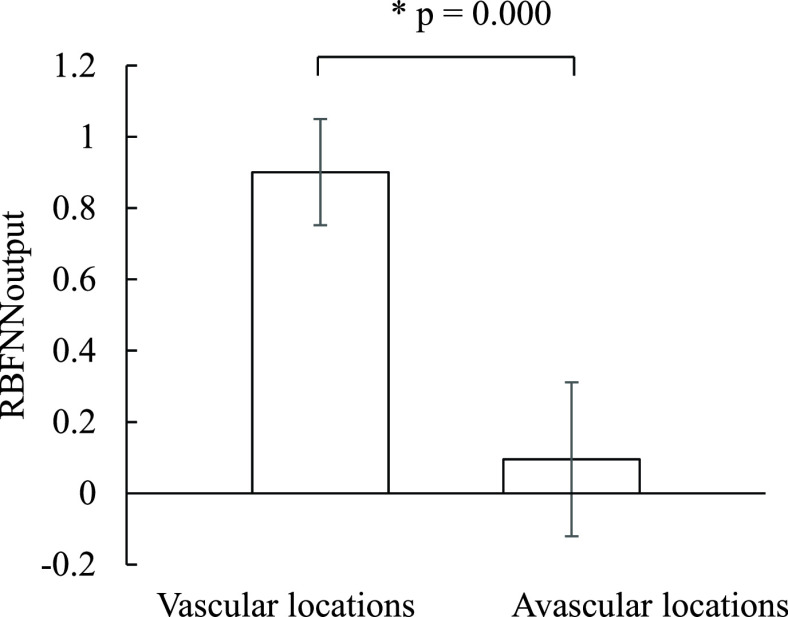
RBFNN outputs for different groups.

In total, 360 data sets (number of vascular groups = 180; number of avascular groups = 180) collect from different tissue in the animal experiment were used for training. Before classification, the number of hidden neurons and optimal threshold had to be determined in the training stage. The number of hidden neurons was set as 32, 64, and 128, and the threshold was set as 0–1. The optimal performance (F-measure = 94.74%, sensitivity = 95%, PPV = 94.48%, and accuracy = 94.72%) was obtained when the number of hidden neurons and threshold were set as 64 and 0.6, respectively. In the blind test stage, 20 data sets (vascular location groups = 10; avascular location groups = 10) were used. Here, the number of hidden neurons and threshold were set as 64 and 0.6, respectively. The experimental results revealed that the proposed system achieved high performance in classifying the two groups (F-measure = 90%, sensitivity = 90%, PPV = 90%, and accuracy = 90%). Fig. 7 shows the RBFNN outputs for different groups; the RBFNN output for vascular groups (0.901 ± 0.149) was significantly higher than that for avascular groups (0.095 ± 0.216; p = 0.000).

**FIGURE 8.**
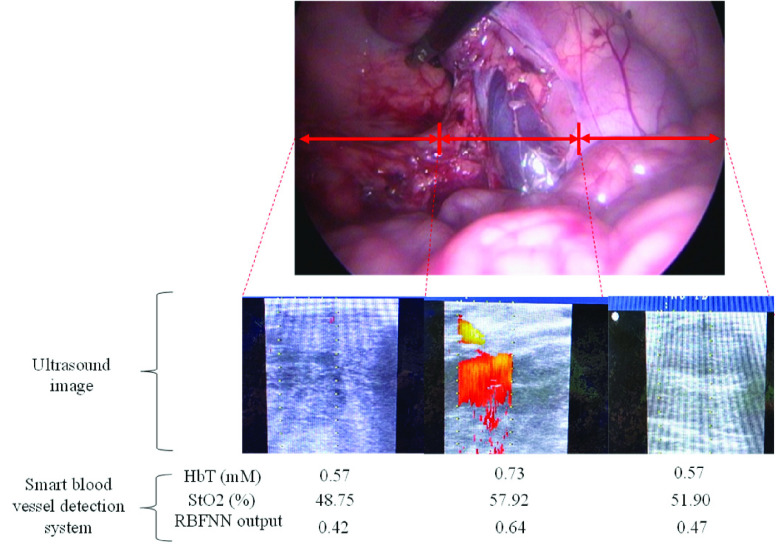
Ultrasound image and hemoglobin parameters of the proposed system in the vivo experiment.

Fig. 8 shows the detection results for different locations in the abdominal cavity in the experiment on piglets. An ultrasonic instrument (Flex focus 8001202, BK medical, Denmark) and a robotic drop-in transducer (Type8826, BK medical, Denmark) were used to obtain ultrasound images at the same locations. The HbT and StO_2_ values in the middle locations of the abdominal cavity for optical pairs 1–3 were higher than those at the side locations. Moreover, the RBFNN outputs for the middle locations (0.639 ± 0.059) were higher than those for the right side (0.468 ± 0.000) and left side (0.423 ± 0.021). In the ultrasound image, the color part represents the effluence or influx state of bodily fluid, indicating blood flow. Therefore, the middle location may contain blood vessels.

## IV. Discussions

As shown in Fig. 5 (b), the HbT and StO_2_ values at the locations above the plastic tube were higher than those at other locations, and the experimental results agreed favorably with the absorption spectra for a plastic tube containing red ink. The higher HbT and StO_2_ values at the 5-mm-deep simulated blood vessel, shown in Fig. 5 (c), were estimated by optical pair 1 of the optical probe. For optical pair 3 of the optical probe, all HbT and StO_2_ values at the simulated blood vessels at depths of 5, 10, and 15 mm were higher because the penetration depth of the light was approximately half the distance between the light source and detector. When the distance between the light source and detector is greater, the light must pass through the shallow layer to the deeper layer so that absorbance information can be obtained for different layers. The HbT and StO_2_ values for arteries were higher than those for veins (Fig. 6), indicating that the volume of oxygenated blood in arteries was higher than that in veins, which accurately reflects human physiology. Moreover, the changes in optical density for 700- and 910-nm-wavelength light were respectively slightly lower and much lower for veins than for arteries (Fig. 6). However, for 700- and 910-nm-wavelength light, the absorbance of deoxyhemoglobin was higher and lower, respectively, than that of oxyhemoglobin. Therefore, the change in optical density at a specific wavelength was caused by not only absorbance but also blood volume. The changes in the 910-nm-wavelength optical density for arteries and veins were greater than those for skin and muscle; this may have been due to the higher absorption coefficient of arteries and veins compared with those of skin and muscle [22], [23]. The StO_2_ value of the liver was much lower than that of other tissues. The liver contains oxyhemoglobin, deoxyhemoglobin, and bile [24]. Bile has a specific absorption spectrum [25] and may influence the estimation of StO_2_. The HbT and StO_2_ values at vascular locations can be distinguished from those at avascular locations because blood vessels contain more blood than other human tissues, such as skin and muscle.

Several approaches have been proposed for detecting blood vessels; their comparison is summarized in Table 1. The vein visualization device [7] is generally used for screening the skin’s surface. The device emits near-infrared light (wavelength: 785 nm) and receives the light reflected from the skin’s surface. However, this device is unsuitable for laparoscopic surgery due to its shallow measuring depth. In clinical settings, the Doppler ultrasound system can be used for detecting blood flow and may be employed in laparoscopic surgery [26]. Such systems determine the frequency difference between transmitted and reflected ultrasound signals and estimate the blood flow rate to recognize vascular locations. However, the tip direction of the ultrasound probe is difficult to control in body cavities, and the blood flow estimation is strongly affected by the angle and certain body tissue barriers such as bones. Optical coherence tomography (OCT) [27] can also be used to obtain high-resolution images of tissue structure. However, the OCT technique can only provide information on tissue structure; the identification of arteries and veins requires the expertise of physicians. Moreover, the measuring depth of the OCT system is relatively small (~1.5 mm). In contrast to these methods, the rotatable stick in the proposed system is easy for a physician to operate in a body cavity, and the angle of the optical probe is easily adjusted to navigate rough tissue surfaces. Moreover, the specific LED-PD placement of the optical probe helps in detecting blood vessels at different depths simultaneously. Using the difference between HbT and StO_2_ values of different tissues, vascular and avascular locations can be successfully distinguished using a neural network. Although the resolution of this system is lower than that of the aforementioned systems, the system offers a greater measuring depth (~1.5–2 cm).

**TABLE 1:** System Comparison Between Proposed System and Other Systems

	Vein visualization device [7]	Doppler ultrasound [26]	SD-OCT 5000 [27]	Proposed system
Sensing technique	Near-infrared spectroscopy	Ultrasound	Optical coherence tomography	Near infrared spectroscopy
Sensor type	CCD camera	Ultrasound Probe	OCT probe	Optical probe
Transmission mode	–	USB	USB	Bluetooth
System size (cm^3^)	$5\times 6 \times 20$	$51.9\times 59.5 \times 160$	$65\times 46 \times 53$	$5.3\times 4\times 1.8$
Wavelength of light source (nm)	785	–	840	700, 910
Analysis parameters	2-D image	2-D image	3-D image	Hemoglobin parameters and optical density
System complexity	Low	High	High	Low
Applications	Vein visualization	Laparoscopy surgery	Eye examination	Laparoscopy surgery
Advantages	Detail information of blood vessel image, easy operation, and lower cost	Deeper measuring depth	Higher-resolution image	Multi-depths measurement, Good distinguishability of tissue types, easy operation, and lower cost
Limitations	Lack of multi-depths measurement, and shallower measuring depth	Low distinguishability of tissue type, influence of measuring angle and bone, requirement of experienced operator, and higher cost	Low distinguishability of tissue type, shallower measuring depth, requirement of experienced operator, and higher cost	Lower spatial resolution

## V. Conclusion

In this study, a smart blood vessel detection system for laparoscopic surgery was proposed. Using the differences in HbT and $StO_{2}$ values between different tissues, vascular and avascular locations can be successfully recognized. Moreover, the specific LED-PD placement of the optical probe helps in detecting blood vessels at different depths simultaneously. The proposed system was validated using phantom and animal experiments. The changes in the optical density at specific wavelengths and the HbT and $StO_{2}$ values of various tissues—the skin, veins, arteries, the liver, and muscle—were significantly different. The proposed system can effectively distinguish vascular and avascular locations by using a neural network. The proposed system offers the advantages of low cost, easy operation, excellent ability to recognize various types of tissue, and multiple measuring depths. Therefore, the proposed system can be regarded as a favorable system prototype for vessel detection in laparoscopic surgery and may assist physicians in preventing damage to blood vessels during surgery.
